# Phylogenomics of *Acinetobacter* species and analysis of antimicrobial resistance genes

**DOI:** 10.3389/fmicb.2023.1264030

**Published:** 2023-10-19

**Authors:** Antonella Migliaccio, James Bray, Michele Intoccia, Maria Stabile, Giovanni Scala, Keith A. Jolley, Sylvain Brisse, Raffaele Zarrilli

**Affiliations:** ^1^Department of Public Health, University of Naples “Federico II”, Naples, Italy; ^2^Department of Biology, University of Oxford, Oxford, United Kingdom; ^3^Department of Biology, University of Naples Federico II, Naples, Italy; ^4^Department of Chemical Sciences, University of Naples Federico II, Naples, Italy; ^5^Institut Pasteur, Université Paris Cité, Biodiversity and Epidemiology of Bacterial Pathogens, Paris, France

**Keywords:** *Acinetobacter baumannii* group, *Acinetobacter* spp., Pasteur Multilocus Sequence Typing, ribosomal Multilocus Sequence Typing, maximum-likelihood phylogeny, antimicrobial resistance genes

## Abstract

**Introduction:**

Non-*baumannii Acinetobacter* species are increasingly isolated in the clinical setting and the environment. The aim of the present study was to analyze a genome database of 837 *Acinetobacter* spp. isolates, which included 798 non-*baumannii Acinetobacter* genomes, in order to define the concordance of classification and discriminatory power of 7-gene MLST, 53-gene MLST, and single-nucleotide polymorphism (SNPs) phylogenies.

**Methods:**

Phylogenies were performed on Pasteur Multilocus Sequence Typing (MLST) or ribosomal Multilocus Sequence Typing (rMLST) concatenated alleles, or SNPs extracted from core genome alignment.

**Results:**

The Pasteur MLST scheme was able to identify and genotype 72 species in the *Acinetobacter* genus, with classification results concordant with the ribosomal MLST scheme. The discriminatory power and genotyping reliability of the Pasteur MLST scheme were assessed in comparison to genome-wide SNP phylogeny on 535 non-*baumannii Acinetobacter* genomes assigned to *Acinetobacter pittii*, *Acinetobacter nosocomialis*, *Acinetobacter seifertii*, and *Acinetobacter lactucae* (heterotypic synonym of *Acinetobacter dijkshoorniae*), which were the most clinically relevant non-*baumannii* species of the *A. baumannii* group. The Pasteur MLST and SNP phylogenies were congruent at Robinson-Fould and Matching cluster tests and grouped genomes into four and three clusters in *A. pittii*, respectively, and one each in *A. seifertii*. Furthermore, *A. lactucae* genomes were grouped into one cluster within *A. pittii* genomes. The SNP phylogeny of *A. nosocomialis* genomes showed a heterogeneous population and did not correspond to the Pasteur MLST phylogeny, which identified two recombinant clusters. The antimicrobial resistance genes belonging to at least three different antimicrobial classes were identified in 91 isolates assigned to 17 distinct species in the *Acinetobacter* genus. Moreover, the presence of a class D oxacillinase, which is a naturally occurring enzyme in several *Acinetobacter* species, was found in 503 isolates assigned to 35 *Acinetobacter* species.

**Conclusion:**

In conclusion, Pasteur MLST phylogeny of non-*baumannii Acinetobacter* isolates coupled with *in silico* detection of antimicrobial resistance makes it important to study the population structure and epidemiology of *Acinetobacter* spp. isolates.

## Introduction

*Acinetobacter* spp. are aerobic, non-fermentative, Gram-negative coccobacilli that are widely distributed in the environment and are responsible for infections in animals and humans (Bouvet and Grimont, 1986; Cosgaya et al., 2017; Wong et al., 2017; Cools et al., 2019). The genus *Acinetobacter* includes 77 child taxa with a validly published and correct name (https://www.bacterio.net/genus/acinetobacter; accessed on June 2023). As the identification of *Acinetobacter* isolates at the species level has been difficult to obtain using standard phenotypic methods (Bouvet and Grimont, 1986; Cools et al., 2019), Matrix-Assisted Laser Desorption-Ionization-Time of Flight (MALDI-TOF) mass spectrometry (Marí-Almirall et al., 2017; Šedo et al., 2018) or genotypic methods, which use partial *rpoB* sequencing (Gundi et al., 2009) or ribosomal MLST analysis (Jolley et al., 2012), have been applied for correct *Acinetobacter* species assignment. The above techniques have identified *Acinetobacter baumannii* as the most clinically relevant species of the *Acinetobacter* genus, which has been demonstrated to cause community and healthcare-associated infections (Wong et al., 2017; Whiteway et al., 2022). Genomic epidemiology of *A. baumannii* isolates has shown the global spread of distinct clonal lineages, which have been selected because of their resistance to a broad range of antimicrobials, including carbapenems (Wong et al., 2017) and have been responsible for epidemics worldwide (Gaiarsa et al., 2019; Hamidian and Nigro, 2019). In addition to *A. baumannii*, *A. nosocomialis*, *A. pittii*, *A. seifertii*, and *A. lactucae* (formerly identified as *A. dijkshoorniae*) have been increasingly isolated from humans and reported to be responsible for infections (Cosgaya et al., 2017). *A. baumannii*, *A. nosocomialis*, *A. pittii*, *A. seifertii*, and *A. lactucae* showed closely related phenotypic and genotypic features and were considered members of the *A. baumannii* group (Cosgaya et al., 2017; Marí-Almirall et al., 2017). Epidemics caused by multidrug-resistant (MDR) and carbapenem-resistant *A. nosocomialis*, *A. pittii*, and *A. seifertii* have been increasingly reported (Chen et al., 2018, 2019; Chopjitt et al., 2021; Li et al., 2021).

The present study aimed to perform phylogenomic analysis of 837 isolates assigned to 72 distinct species in the *Acinetobacter* genus using the Pasteur MLST scheme, compare phylogenetic congruence with genome-based and ribosomal MLST (rMLST)-based phylogenies of *A. baumannii* group genomes, and identify antimicrobial resistance genes in *Acinetobacter* spp. genomes.

## Materials and methods

### Genome dataset

Bacterial genomes included in the analysis were manually selected from the PubMLST database^1^ until January 2022. In detail, we selected 39 *A. baumannii* complete genomes assigned to international clonal lineages ICI, ICII, and ICIII, which corresponded to Pasteur ST1, ST2, and ST3, respectively, and to additional epidemic clonal lineages assigned to Pasteur ST10, ST25, ST32, ST52, ST78, and ST79 (Gaiarsa et al., 2019). Furthermore, we collected from the National Center for Biotechnology Information (NCBI) high-quality complete genomes and, when not available, scaffolded sequences of all species into the *Acinetobacter* genus. The “parameters” to consider the genomes of “high quality” were: N50 ≥ 10,000 bp; number of contigs ≤1,000; identification of the correct species through rMLST (Jolley et al., 2012). The *Acinetobacter* selected genomes were typed by *A. baumannii* Pasteur MLST scheme (Diancourt et al., 2010) and rMLST (Jolley et al., 2012) using the BIGSdb software available at https://pubmlst.org/organisms/acinetobacter-baumannii/ (Jolley et al., 2018). The characteristics of the genomes were included in Supplementary Table S1 and available at https://pubmlst.org/bigsdb?db=pubmlst_abaumannii_isolates&page=project&project_id=8.

### Phylogenetic and statistical analyses

The allelic profiles of Pasteur and ribosomal MLST schemes were extracted from all the genomes, and then the sequences were aligned using Muscle (Edgar, 2004) to generate the neighbor-joining trees using the BIGSdb software (Jolley et al., 2018). The core genome single-nucleotide polymorphisms (SNPs) were detected using the tools of PARSNP v1.1.2 (Treangen et al., 2014), and the SNP alignment was performed considering the ascertainment bias using the Lewis correction (Lewis, 2001). In detail, each genome was aligned to the reference genome NC_010611.1 of ACICU, and the alignments were then concatenated using Muscle (Edgar, 2004). The maximum-likelihood phylogenies of 574 genomes belonging to the *A. baumannii* group (*A. baumannii, A. pittii, A. seifertii, A. lactucae*, and *A. nosocomialis*) were performed using the concatenated alleles of the Pasteur and ribosomal MLST schemes and a reference phylogeny using genome-wide data (a core genome of 372 high-quality genes and an alignment of 17,072 SNPs). The phylogenies of Pasteur MLST, ribosomal MLST, and core SNP alignments were inferred through the GTR-GAMMA model at 100 bootstrap replicates using RAxML v.8 (Stamatakis, 2014). GTR’s GAMMA model was used for its ability to optimize the transition/transversion speed ratio and the α parameter of the gamma rate heterogeneity distribution (Stamatakis, 2014). The phylogenetic trees and annotations were visualized using the iTol v6 software.^2^

### Statistical analyses

The statistical analyses were performed using the Robinson-Fould (R-F) and Matching clusters (M-C) topology-based tests employing TreeCmp (Bogdanowicz et al., 2012). The M-C test calculates the number of topological changes that must be made to transform a tree into a reference tree. The R-F test counts the number of splits that are unique to one of the two trees. In both cases, the two analyzed trees are identical if the value is zero. The likelihood-based Shimodaira-Hasegawa (SH) test (Shimodaira and Hasegawa, 1999) was performed with RAXML (Stamatakis, 2014). In this test, a null hypothesis assumes that two compared trees are both a correct interpretation of an alignment. The tested hypothesis is that one or more trees better represent the data. *p*-values lower than 0.05 indicate that the two trees are significantly different.

### Analysis of antimicrobial resistance genes

The antimicrobial resistance genes were detected using abricate^3^ based on the ResFinder 4.0 database (Bortolaia et al., 2020). The presence of the gene and percentage of identity were indicated in the matrix in Supplementary Table S2. The threshold identity of 80%, the minimum length of 80% matches, and the coverage value of more than 90% were selected for each gene. The following acquired resistance genes were analyzed: class A, B, C, D β-lactamase, folate pathway inhibitors, rifampicin, aminoglycoside, chloramphenicol, colistin, macrolide, quaternary ammonium salts, and tetracycline resistance genes (Supplementary Table S2). The multiple alignment and phylogeny of class D β-lactamase genes were performed using Clustal W (Thompson et al., 1994) and RAXML v.8 (Stamatakis, 2014), respectively. The genomes of *Acinetobacter* genus were classified as MDR if they carried antimicrobial-resistant genes for at least three of the nine classes of antimicrobials considered by Magiorakos et al. (2012).

### Clustering of Pasteur MLST and core SNPs phylogenies

The clusters of Pasteur sequence types (STs) were determined using the eBURST algorithm as described previously (Feil et al., 2004). Minimum spanning trees of STs were built with Phyloviz using the goeBURST algorithm (Ribeiro-Gonçalves et al., 2016). Minimum-spanning trees were generated from the seven alleles of each MLST scheme, and species were assigned based on clustering with reference STs. Additionally, SNP analysis by PARSNP was visualized using Gingr (Treangen et al., 2014), which provided an interactive display of multi-alignment variants and a phylogenetic tree estimated from the core genome alignment. Then, the values of the maximum unique matches and the data were evaluated to study the cluster phylogeny of SNPs (Treangen et al., 2014).

## Results

### *Acinetobacter genus* database

**Figure 1:**
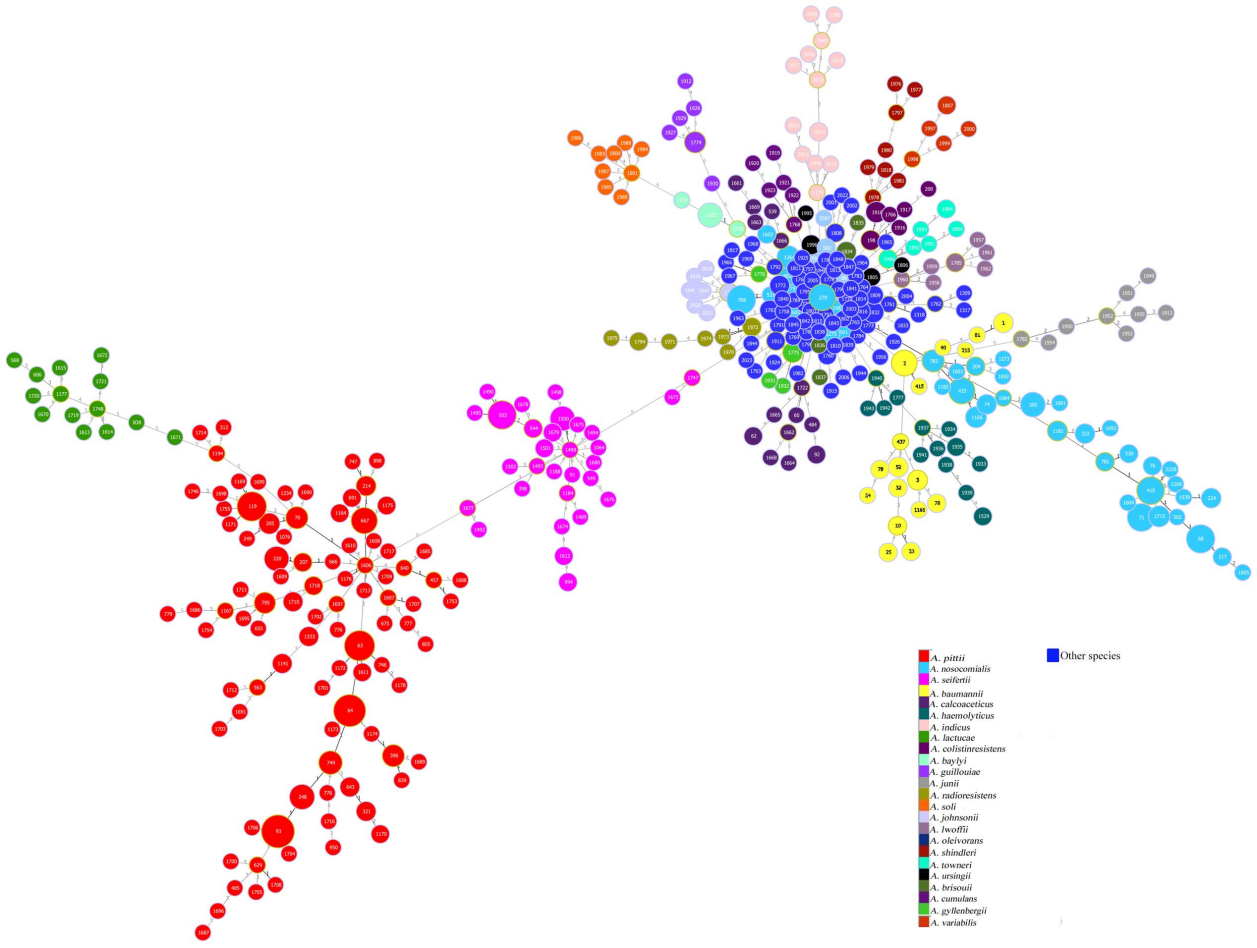
Minimum spanning tree of 837 genomes inferred by the Pasteur MLST scheme. The different colors indicate the 24 *Acinetobacter* species that are represented by more than 6 isolates, while the blue color indicates the 48 *Acinetobacter* species that are represented by less than 6 genomes. The numbers within each circle indicate the ST. The size of the circle is proportional to the number of genomes belonging to the same ST. The figure was obtained using the eBURST algorithm with the Phyloviz software (Ribeiro-Gonçalves et al., 2016).

The database consisted of 837 genomes assigned to 72 distinct species in the *Acinetobacter* genus, which were identified at genus and species levels using the ribosomal MLST scheme (Jolley et al., 2012) and showed a genome size range of 2.85–4.85 Mpb (Supplementary Table S1). The Pasteur MLST scheme (Diancourt et al., 2010) was able to assign an allelic profile and an ST to all 837 genomes belonging to 72 species in the *Acinetobacter* genus; while, the ribosomal MLST scheme assigned an ST to 806 out of 837 genomes because from 1 to 20 alleles could not be identified in the genomes belonging to *A. baumannii, A. nosocomialis, A. bereziniae, A. pittii, A. radioresistens*, and *A. seifertii*. In addition, the rMLST scheme identified paralogues in 11 out of 53 loci paralogous in 57 *Acinetobacter* spp. (Supplementary Table S1). The genome-wide distances of the whole database analyzed using the minimum spanning tree (MST) with the Pasteur MLST scheme showed that the most abundant species were *A. pittii* (*n* = 282), *A. nosocomialis* (*n* = 175), *A. seifertii* (*n* = 61), *A. baumannii* (*n* = 39), and *A. lactucae* (*n* = 14), while 263 genomes were assigned to other 67 species of *Acinetobacter* genus (Figure 1). The 574 genomes assigned to the above five species have been considered the most clinically relevant species and were included in the *A. baumannii* group (Cosgaya et al., 2017). Among genomes within the *A. baumannii* group, the genetically closest species were *A. pittii* with *A. lactucae* or *A. seifertii*, showing 5 and 6 locus variants (LVs) genome-wide distance, respectively. The *A. nosocomialis* genomes assigned to ST782 and to the most frequent ST279 showed 4 and 5 LVs genome-wide distances with respect to CC2 *A. baumannii* genomes, respectively (Figure 1).

### Maximum-likelihood phylogeny of *Acinetobacter baumannii* group

**Figure 2:**
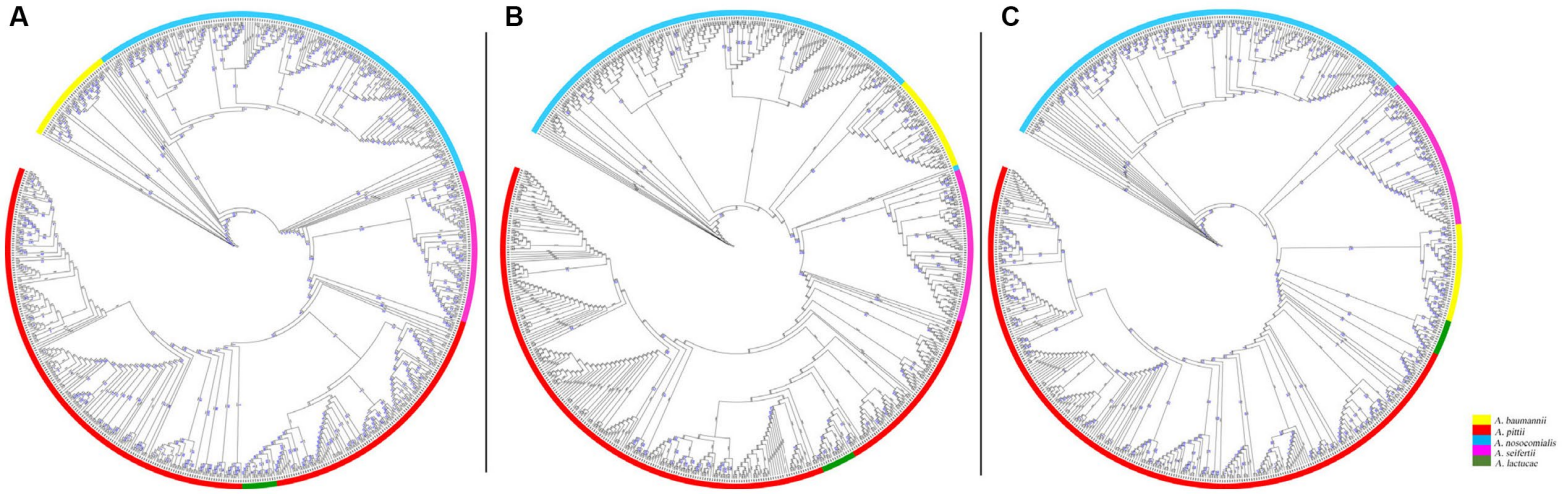
Phylogeny of *A. baumannii* group genomes. Maximum-likelihood phylogeny of 574 *A. baumannii* group genomes, including 39 *A. baumannii* (yellow labels), 285 *A. pittii* (red labels), 175 *A. nosocomialis* (blue labels), 61 *A. seifertii* (pink labels), and 14 *A. lactucae* genomes (green labels), was inferred on coreSNPs **(A)**, Pasteur MLST **(B)**, and rMLST **(C)** using RAxML. Bootstrap values are indicated in blue on tree branches, while the lengths of the branches are indicated in black. The figures were obtained using iTol v6 (https://itol.embl.de/).

The core genome-SNP, Pasteur MLST, and rMLST maximum-likelihood phylogenies of 574 genomes belonging to the *A. baumannii* group (*A. baumannii, A. pittii, A. seifertii, A. lactucae*, and *A. nosocomialis*) showed similar inter- and intra-species distributions of branch lengths and nodes (Figure 2). In particular, the branch lengths of SNPs and rMLST phylogenies had values between ~10^−6^ and ~ 10^−2^ (Figures 2A,C), while the branch lengths of Pasteur MLST phylogeny were between ~10^−6^ and ~ 10^−3^ (Figure 2B). The core genome SNPs and Pasteur MLST phylogenies showed bootstrap values greater than 50 for ancestral nodes of all species belonging to the *A. baumannii* group (Figures 2A,B). In addition, rMLST phylogeny assigned bootstrap values greater than 50 to the ancestral nodes of *A. baumannii, A. seifertii*, and *A. lactucae* species, while bootstrap values of 36 for *A. pittii* ancestral node and more uneven values ranging from 21 to 94 were assigned across *A. nosocomialis* genomes (Figure 2C). The above data demonstrated that all three phylogenies identified *A. baumannii, A. pittii, A. seifertii, A. lactucae*, and *A. nosocomialis* as distinct species in the *A. baumannii* group and a strong genomic similarity between *A. lactucae* and *A. pittii* species. Moreover, all three maximum-likelihood phylogenies showed high genomic heterogeneity among *A. nosocomialis* genomes (Figure 2). The statistical comparison between core genome SNP phylogeny and Pasteur MLST or rMLST phylogenies using the R-F and M-C tests showed similar statistical values and concordance among phylogenies (Table 1).

**Table 1:** Statistical comparison of genome-wide SNPs and MLST schemes phylogenies.

Reference tree	Tree	Robinson-Fould cluster	Matching cluster	SH-test	
				D LH	SD
SNP	Pasteur MLST	475	6143	−414,530,11	91,004,74
SNP	rMLST	447	5493	−15,272,756	34,776,25
Pasteur MLST	rMLST	486	6729	−42,083,157	90,941,15

### Clustering of core SNP and Pasteur MLST phylogenies of *Acinetobacter baumannii* group

**Figure 3:**
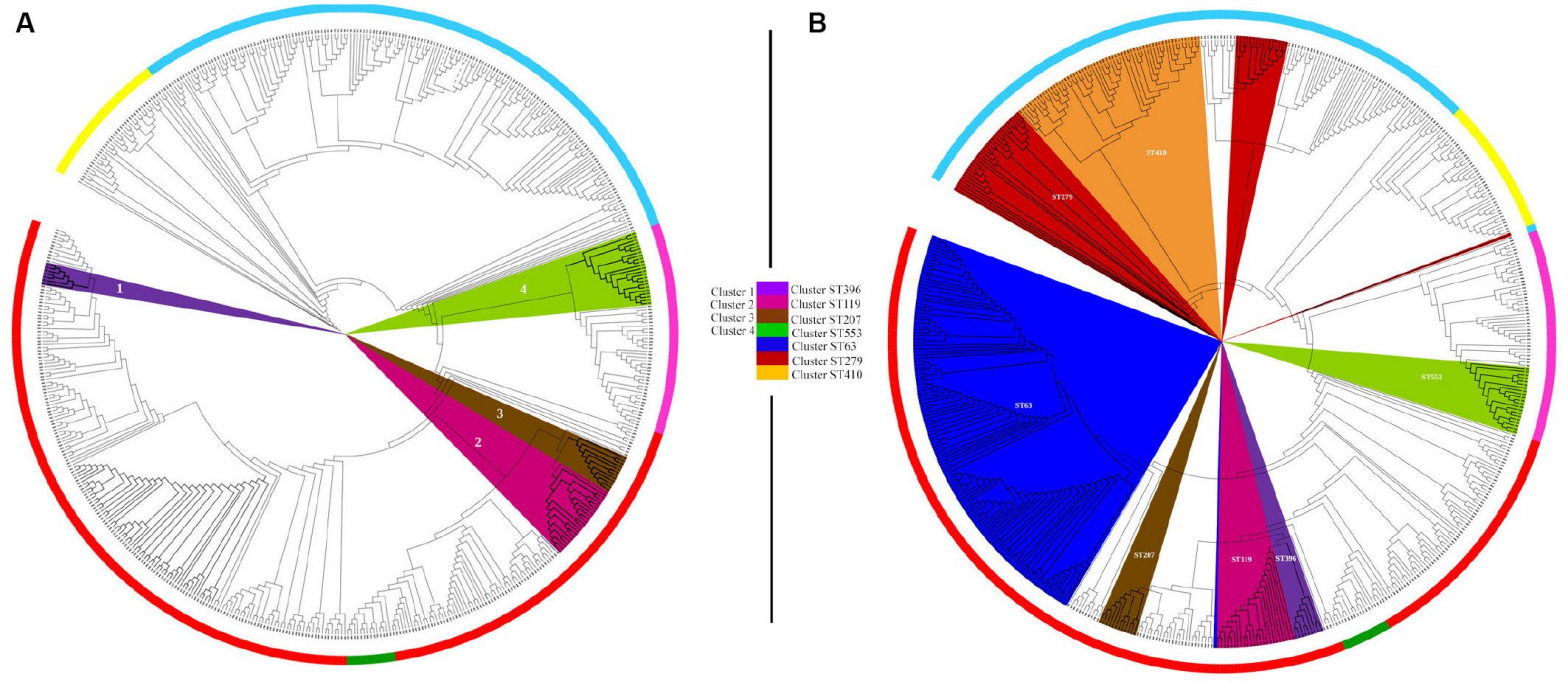
Cluster analysis of core genome SNP and Pasteur MLST phylogenies. Cluster analysis of core genome SNP **(A)** and Pasteur MLST **(B)** phylogenies of 574 genomes belonging to *Acinetobacter baumannii* group. **(A)** The four main clusters identified in the core genome SNP phylogeny are highlighted in violet (cluster 1), fuxia (cluster 2), brown (cluster 3), and green (cluster 4). **(B)** The seven main clusters identified in the Pasteur MLST phylogeny are highlighted in blue (cluster ST63), brown (cluster ST207), pink (cluster ST119), violet (cluster ST296), green (cluster ST553), orange (cluster ST410), and red (cluster ST279). The phylogenies were analyzed with RAxML, and the figures were obtained using iTol v6 (https://itol.embl.de/).

To evaluate the discriminatory power of the core genome SNP phylogeny and the Pasteur MLST phylogeny, we analyzed and compared the clusters identified by the two phylogenies. Clustering of core SNP phylogeny identified three clusters (1–3) in *A. pittii* genomes, one cluster (4) in *A. seifertii* genomes, and no clusters in *A. nosocomialis* genomes (Figure 3A). Cluster analysis of Pasteur MLST phylogeny showed four clusters in *A. pittii* having ST63, ST119, ST207, and ST396 as ancestral STs; two clusters in *A. nosocomialis* with ST410 and ST279 as ancestral STs; and one cluster in *A. seifertii* with ancestral ST553. *Acinetobacter baumannii* genomes showed three clades assigned to ST1, ST2, and ST10; and *A. lactucae* genomes counted 14 singletons and no clades (Figure 3B). Interestingly, *A. pittii* clusters 1, 2, and 3 of the core SNP phylogeny corresponded to *A. pittii* ST396, ST119, and ST207 clusters in the Pasteur MLST phylogeny, respectively; *A. seifertii* cluster 4 of the core SNP phylogeny corresponded to *A. seifertii* ST553 cluster of the Pasteur MLST phylogeny (Figure 3).

### Antimicrobial resistance genes in *Acinetobacter* spp. genomes

**Figure 4:**
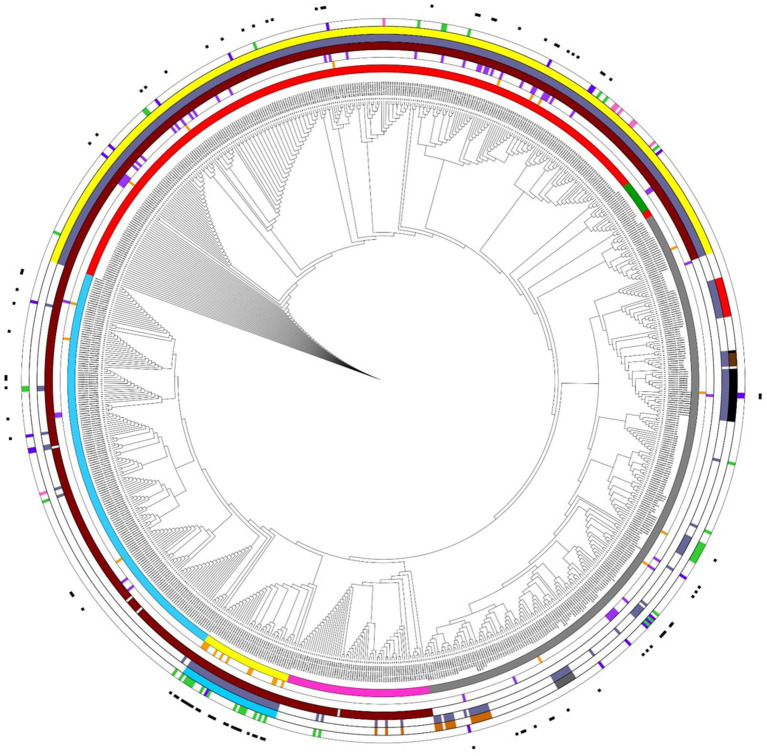
Antimicrobial resistance genes in a neighbor-joining tree of 837 *Acinetobacter* spp. genomes inferred on the Pasteur MLST scheme. The inner ring indicates the following species: *A. baumannii* (yellow labels), *A. pittii* (red labels), *A. nosocomialis* (blue labels), *A. seifertii* (pink labels), *A. lactucae* (green labels), and other 263 *Acinetobacter* spp. genomes (gray labels). The second inner ring (orange) indicates class A β-lactamase. The third inner ring (purple) indicates class B β-lactamase. The fourth inner ring (claret) indicates class C β-lactamase. The fifth inner ring (violet) indicates class D β-lactamase. The two external rings indicate the presence of at least one or two class D β-lactamases using the colored groups shown in Supplementary Figure S1. The external black rectangles indicate the isolates classified as MDR. The figure was obtained using iTol v6 (https://itol.embl.de/).

We analyzed the presence of antimicrobial resistance genes in the 837 genomes assigned to 72 distinct *Acinetobacter* species. The ResFinder software identified 188 genes encoding for resistance to 12 antimicrobial categories (Supplementary Table S2). The presence of class A, B, C, and D β-lactamases in the neighbor-joining tree of the 837 genomes assigned to 72 distinct *Acinetobacter* species is shown in Figure 4. The class A β-lactamase genes *bla*_CARB-8,-2,-14,-16_ were found in 1 *A. nosocomialis,* 4 *A. pittii,* 1 *A. towneri,* and 1 *A. bereziniae* genomes; the class A β-lactamase gene *bla*_*SCO*-1_ was found in 1 *A. radioresistens* genome. In addition, class A β-lactamase genes *bla*_*TEM*-1A,-1B,-1D_ were found in 9 of 39 *A. baumannii* genomes; *bla*_*PER*-1,-2_ ESBL genes were found in 1 *A. baumannii*, 2 *A. nosocomialis*, 1 *A. pittii*, and 1 *A. radioresistens* genomes; *bla*_*VEB*-1,-7_ ESBL genes were found in 1 *A. baumannii* and 2 *A. pittii* genomes. The class B metallo-β-lactamase (MBL) genes *bla*_*GIM*-1_, *bla*_IMP-1,-4,-14,-19,-34_ *bla*_NDM-1,-16_, and *bla*_VIM-2,-4_ were identified in 12 of 72 species of *Acinetobacter* genus. Among species, 34 of 282 *A. pittii* genomes showed at least one MBL gene. The *bla*_*NDM*-1_ was the most frequent MBL gene and was found in *A. nosocomialis, A. pittii, A. lactucae, A. junii, A. bereziniae, A. cumulans, A. wuhouensis, A. sichuanensis, A. rongchengensis, A. indicus,* and *A. variabilis* genomes. The *bla*_*ADC*-25_ class C β-lactamase was found in the five species belonging to *A. baumannii* group and *A. calcoaceticus* genomes, but not in other *Acinetobacter* spp. (Figure 4; Supplementary Table S2). The class D oxacillinase, which is a naturally occurring enzyme in several *Acinetobacter* species, was found in 503 isolates assigned to 35 *Acinetobacter* species (Figure 4; Supplementary Table S2). In all, 94 class D β-lactamase genes belonging to 11 distinct *bla*_OXA_ family genes (*bla*_OXA-211_, *bla*_OXA-134_, *bla*_OXA-214_, *bla*_OXA 294_, *bla*_OXA-51_, *bla*_OXA-213_, *bla*_OXA-274_, *bla*_OXA-286_, *bla*_OXA-58_, *bla*_OXA-40_, and *bla*_OXA-23_) were identified (Supplementary Figure S1). Among the five species of *A. baumannii* group, *A. baumannii*, *A. pittii*, and *A. lactucae* genomes showed intrinsic class D β-lactamase belonging to *bla*_OXA-51_ and *bla*_OXA-213_ family genes, respectively, while *A. nosocomialis* and *A. seifertii* genomes did not show any intrinsic class D β-lactamase. Similarly, intrinsic class D β-lactamases were identified in other *Acinetobacter* species, such as *bla*_OXA-134-like_ in *A. lwoffii* and *A. schindleri*, *bla*_OXA-211_ family in *A. johnsonii*, *bla*_OXA-213_ family in *A. calcoaceticus*, *bla*_OXA-214_ family in *A. haemolyticus*, *bla*_OXA-228_ family in *A. bereziniae*, *bla*_OXA-286_ family in *A. viviani, A. disperses*, and *A. courvalini* genomes, and *bla*_*OXA*-294_ family gene in *A. proteoliticus, A. gyllenbergii,* and *A. colistinresistens* (Figure 4). Also, the presence of class D carbapenemase genes belonging to *bla*_OXA-23_, *bla*_OXA-40_, or *bla*_OXA-58_ family genes were found in 16 of 39 *A. baumannii* genomes, 27 of 282 *A. pittii* genomes, 8 of 145 *A. nosocomialis* genomes, 2 of 2 *A. seifertii* genomes, 9 of 9 *A. radioresistens* genomes, 5 of 6 *A. cumulans* genomes, and 3 of 11 *A. colistinresistens* genomes (Figure 4). Moreover, 46 of 72 *Acinetobacter* species showed at least one antimicrobial resistance gene (Supplementary Table S2), and 91 isolates assigned to 17 species of the *Acinetobacter* genus showed at least three genes encoding for resistance to three different antimicrobial classes and were classified as MDR isolates (Magiorakos et al., 2012) (Supplementary Table S2; Figure 4).

## Discussion

*Acinetobacter baumannii* frequently causes healthcare-associated infections and is considered the most relevant clinical species of the *Acinetobacter* genus (Wong et al., 2017; Cools et al., 2019). Moreover, non-*baumannii Acinetobacter* species, such as *A. nosocomialis*, *A. pittii*, *A. seifertii*, and *A. lactucae*, showing phenotypic and genotypic characteristics similar to those of *A. baumannii* and included in the *A. baumannii* group, are increasingly reported as responsible for infections in humans (Cosgaya et al., 2017). Furthermore, novel taxa are currently isolated into the *Acinetobacter* genus (https://www.bacterio.net/genus/acinetobacter; accessed on June 2023), which are difficult to identify using standard phenotypic (Cools et al., 2019) and molecular techniques (Marí-Almirall et al., 2017). The present study analyzed the genomic features of 837 isolates assigned to 72 distinct species in the *Acinetobacter* genus. Our data demonstrated that the rMLST and Pasteur MLST schemes were able to genotype and identify at species levels isolates assigned to 72 distinct species in the *Acinetobacter* genus, thus providing useful and validated tools for the identification and characterization of *Acinetobacter* spp. genomes.

In addition, we analyzed the phylogeny of non-*baumannii* genomes of the *A. baumannii* group using core genome SNPs, or concatenated alleles of the Pasteur MLST and rMLST schemes, and compared clusters identified by core genome SNP and Pasteur MLST phylogeny. In keeping with previous data (Gaiarsa et al., 2019), core genome SNP phylogeny and Pasteur MLST phylogeny concordantly identified distinct clades and clonal lineages in *A. baumannii* genomes. The phylogenies of *A. pittii* genomes showed clusters 1, 2, and 3 identified by core genome SNPs, which corresponded to ST396, ST119, and ST207 clusters identified by the Pasteur MLST scheme, respectively, and ST63 cluster identified only by the Pasteur MLST scheme. This is in agreement with previous data showing the prevalence of ST119, ST63, and ST207 genotypic profiles among *A. pittii* isolates (Yang et al., 2012; Kamolvit et al., 2015; Sung et al., 2015; Zhang and Zhou, 2018; Chopjitt et al., 2021) and the identification of a monophyletic bacterial population and distinct clusters among *A. pittii* genomes (Chopjitt et al., 2021). Our data also showed that core genome SNP, Pasteur MLST, and rMLST-based phylogenies all included *A. lactucae* genomes into *A. pittii* genomes and identified *A. lactucae* genomes as singletons. Although *A. lactucae* (formerly *A. dijkshoorniae*) was identified as a distinct species of the *Acinetobacter* genus (Cosgaya et al., 2017; Marí-Almirall et al., 2017), maximum-likelihood phylogenies indicate that *A. lactucae* genomes cannot be distinguished from *A. pittii* genomes. Furthermore, core genome SNP phylogeny of *A. seifertii* genomes identified one single cluster (cluster 4) corresponding to Pasteur ST553, which emerged as a dominant clonal lineage in Asia (Li et al., 2021). As for *A. nosocomialis* genomes, no clades were identified by core genome SNP phylogeny, while one prevalent clade, ST410, was identified by Pasteur MLST phylogeny. This finding is in agreement with previous studies showing the selection of the ST410 genotype among *A. nosocomialis* epidemics (Chen et al., 2018, 2019; Jing et al., 2022). The data shown in this study are also in agreement with previous data showing that the population structure of *A. nosocomialis* genomes is highly heterogeneous (Jing et al., 2022).

The spread of epidemic *A. baumannii* clonal lineages has been favored by their carbapenem resistance and multidrug resistance (Zarrilli et al., 2013; Gaiarsa et al., 2019; Hamidian and Nigro, 2019). Likewise, *A. nosocomialis*, *A. pittii*, *A. lactucae,* and *A. seifertii* responsible for epidemics are carbapenem-resistant and MDR (Cosgaya et al., 2017; Wong et al., 2017). The analysis of antimicrobial resistance genes in the genomes of the 72 species of the *Acinetobacter* genus confirmed the presence of carbapenem resistance genes in *A. baumannii*, *A. nosocomialis*, *A. pittii,* and *A. seifertii* genomes belonging to *A. baumannii* group, while only 1 of 14 *A. lactucae* genomes carried *bla*_NDM-1_ carbapenemase and several antimicrobial resistance genes. In keeping with previous publications (Evans and Amyes, 2014; Cosgaya et al., 2017), the data reported herein showed that *A. baumannii*, *A. lactucae,* and *A. pittii*, but not *A. nosocomialis* and *A. seifertii*, were the species included in the *A. baumannii* group possessing a naturally occurring oxacillinase. The presence of class D beta-lactamases was found in 32 additional species of *Acinetobacter* genomes, thus reinforcing the evidence that this is a characteristic of the *Acinetobacter* genus (Evans and Amyes, 2014). In agreement with previous studies (Cosgaya et al., 2017; Hamidian and Nigro, 2019), we found the presence of *bla*_*OXA*-23_, *bla*_OXA-40_, and *bla*_OXA-58_ carbapenemase genes in *A. baumannii*, *A. nosocomialis*, *A. pittii*, *A. seifertii,* and other *Acinetobacter* species. Notably, all nine *A. radioresistens* genomes in our database showed the *bla*_OXA-23_ gene, which reinforced the evidence that *A. radioresistens* is the progenitor of the *bla*_OXA-23_ gene and the source of carbapenem resistance for *A. baumannii* (Poirel et al., 2008). Moreover, in agreement with previous data showing that the *bla*_NDM_ type is the most common type of metallo-beta-lactamase contributing to carbapenem resistance in clinical isolates of *A. baumannii* (Zarrilli et al., 2013; Gaiarsa et al., 2019) and other *Acinetobacter* spp. (Yang et al., 2012; Yamamoto et al., 2013; Sung et al., 2015; Pfeifer et al., 2020; Alattraqchi et al., 2021), we found *bla*_NDM_ type genes in the genomes of 12 species of the *Acinetobacter* genus.

## Conclusion

The data presented herein analyze a genome collection of isolates assigned to 72 distinct species of *Acinetobacter* genus. The non-*baumannii Acinetobacter* genomes database, which has been validated by rMLST and Pasteur MLST, represents a useful tool for genome sequencing-based identification at the species level and typing of *Acinetobacter* spp. isolates.

The phylogenies of *A. nosocomialis*, *A. lactucae*, *A. pittii*, and *A. seifertii* genomes belonging to the *A. baumannii* group demonstrate the presence of distinct clades in the *A. pittii* and *A. seifertii* genomes, while the *A. nosocomialis* genomes are highly heterogeneous. “*In silico*” analysis of antimicrobial resistance in isolates assigned to 72 distinct species of the *Acinetobacter* genus shows the presence of carbapenemases and resistance genes to several antimicrobial classes in *A. baumannii*, *A. nosocomialis*, *A. pittii*, *A. seifertii*, and other *Acinetobacter* spp.

Pasteur MLST phylogeny of non-*baumannii Acinetobacter* isolates coupled with *in silico* detection of antimicrobial resistance is important to study the population structure and epidemiology of *Acinetobacter* spp. isolates.

## Data availability statement

The datasets presented in this study can be found in online repositories. The names of the repository/repositories and accession number(s) can be found in the article/Supplementary material.

## Author contributions

AM: Formal analysis, Writing – original draft, Data curation, Investigation, Methodology. JB: Data curation, Formal analysis, Writing – original draft, Software. MI: Software, Methodology, Writing – original draft. MS: Formal analysis, Investigation, Writing – original draft. GS: Methodology, Software, Writing – original draft. KJ: Methodology, Conceptualization, Data curation, Formal analysis, Writing – review & editing. SB: Conceptualization, Formal analysis, Methodology, Writing – review & editing. RZ: Conceptualization, Formal analysis, Funding acquisition, Supervision, Writing – original draft, Writing – review & editing.

## Footnotes

^1^ https://pubmlst.org/organisms/acinetobacter-baumannii/

^2^ https://itol.embl.de/

^3^ https://github.com/tseemann/abricate
