# Supplementary figures and images for: Phylogenomics of Acinetobacter species and analysis of antimicrobial resistance genes
Source: Front Microbiol. 2023 Oct 19;14:1264030. doi: 10.3389/fmicb.2023.1264030 (PMC10620307; doi:10.3389/fmicb.2023.1264030)

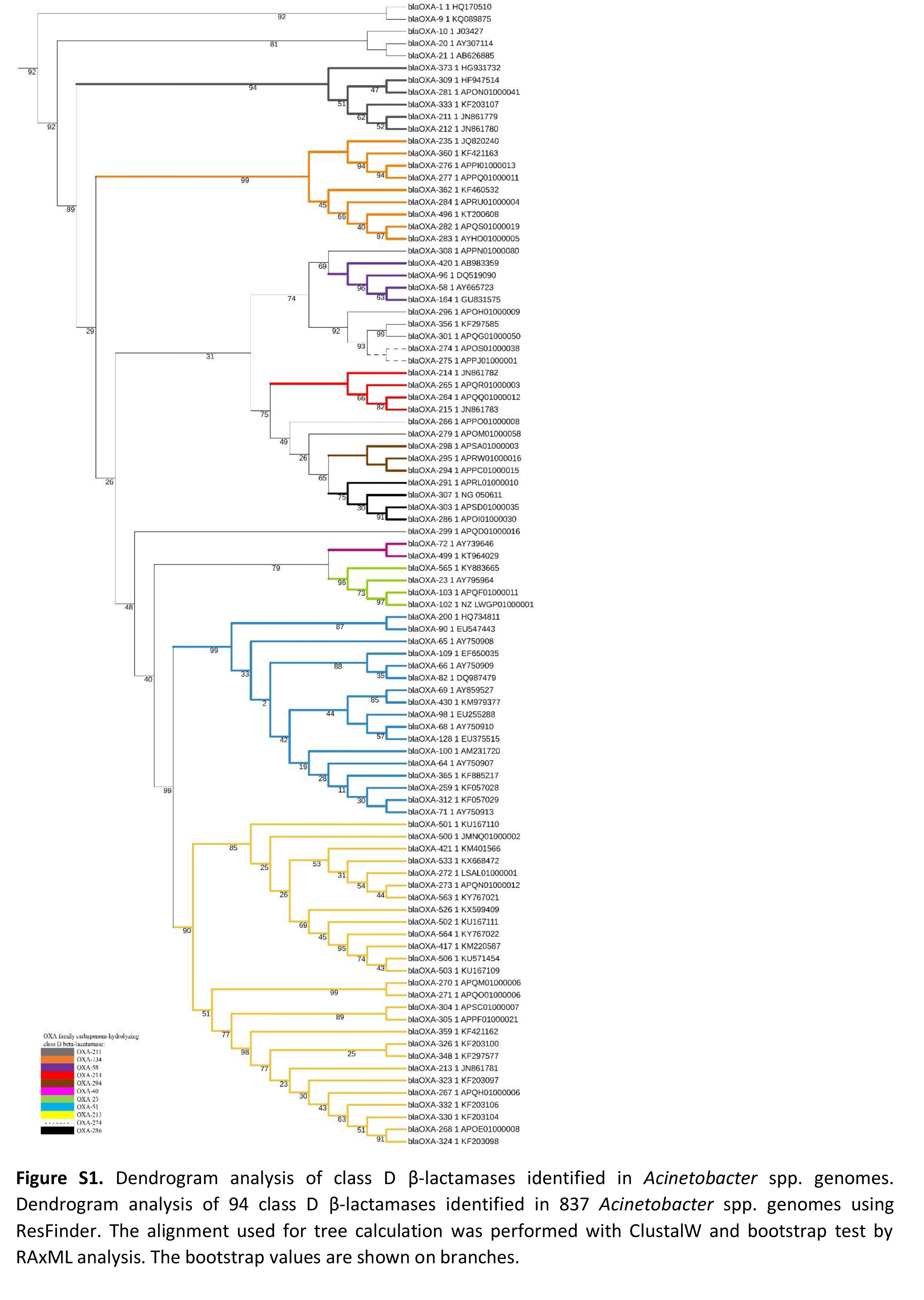

Supplement: Supplementary file 3 [file Image_1.jpg]
